# The clinical-familial correlates and naturalistic outcome of panic-disorder-agoraphobia with and without lifetime bipolar II comorbidity

**DOI:** 10.1186/1744-859X-7-23

**Published:** 2008-11-13

**Authors:** Cristina Toni, Giulio Perugi, Franco Frare, Giuseppe Tusini, Konstantinos N Fountoulakis, Kareen K Akiskal, Hagop S Akiskal

**Affiliations:** 1 Institute of Behavior Sciences 'G. De Lisio', Carrara, Italy; 2 Department of Psychiatry, Neurobiology, Pharmacology and Biotechnologies, Psychiatry Section, University of Pisa, Italy; 3 Adults Mental Health Unit, Pistoia Zone, Pistoia, Italy; 4 Third Department of Psychiatry, Aristotle University of Thessaloniki, Greece; 5 French Depressive and Manic-depressive Association, Rennes, France; 6 International Mood Center, University of California at San Diego, San Diego, CA, USA

## Abstract

**Background:**

Much of the literature on panic disorder (PD)-bipolar disorder (BP) cormorbidity concerns BP-I. This literature emphasizes the difficulties encountered in pharmacologic treatment and outcome when such comorbidity is present. The present report explores these issues with respect to BP-II.

**Methods:**

The sample comprised 326 outpatients (aged 34.5 ± 11.5 years old; 222 females) with Diagnostic and Statistical Manual of Mental Disorders 3rd edn, revised (DSM-III-R) PD-agoraphobia; among them 52 subjects (16%) were affected by lifetime comorbidity with BP-II. Patients were evaluated by means of the Structured Clinical Interview for DSM-IV (SCID), the Panic-Agoraphobia Interview, and the Longitudinal Interview Follow-up Examination (Life-Up) and treated according to routine clinical practice at the University of Pisa, Italy, for a period of 3 years. Clinical and course features were compared between subjects with and without BP-II. All patients received the clinicians' choice of antidepressants and, in the case of the subsample with BP-II, mood stabilizers (for example, valproate, lithium) were among the mainstays of treatment.

**Results:**

In comparison to patients without bipolar comorbidity, those with BP-II showed a significantly greater frequency of social phobia, obsessive-compulsive disorder, alcohol-related disorders, and separation anxiety during childhood and adolescence. Regarding family history, a significantly greater frequency of PD and mood disorders was present among the BP-II. No significant differences were observed in the long-term course of PD or agoraphobic symptoms under pharmacological treatment or the likelihood of spontaneous pharmacological treatment interruptions.

**Conclusion:**

Although the severity and outcome of panic-agoraphobic symptomatology appear to be similar in patients with and without lifetime bipolar comorbidity, the higher number of concomitant disorders in our PD patients with BP-II does indicate a greater complexity of the clinical picture in this naturalistic study. That such complexity does not seem to translate into poorer response and outcome in those with comorbid soft bipolarity probably reflects the fact that we had brought BP-II under control with mood stabilizers. We discuss the implications of our findings as further evidence for the existence of a distinct anxious-bipolar diathesis.

## Background

Frequent comorbidity between panic disorder (PD) and mood disorders has been widely reported in clinical [1-6] and epidemiological studies [7-14]. Previously, most of this research has been essentially limited to the co-occurrence of PD and unipolar disorders; recently, an increasing attention has been paid to the co-occurrence of PD and bipolar disorders (BP) [1,4,15-20].

Analyzing data from the Epidemiological Catchment Area (ECA) study, Chen and Dilsaver [8] reported that the lifetime prevalence of PD was 20.8% in individuals with BP, compared with 10% in those with unipolar major depression, and 0.8% in the general population. In the National Comorbidity Survey [11], the reported risk of comorbid PD is higher in bipolar than in unipolar disorder (odd ratios respectively 11.0 vs 7.0). Similar data, including high rates of PD-BP-II comorbidity, has emerged in epidemiological studies conducted in European populations [14,21,22].

In clinical studies 13% to 23% of adults with PD were found to have comorbid BP [19,23-25]. Conversely, in BP patients, rates of comorbid PD range from 10% to 80% [1,8,16,20,24]. The majority of information on PD comorbidity in patients with BP has been drawn from BP-I samples [10,26-29]. Only a few studies focused on BP-II disorder [18,30]. This relative neglect of PD-BP-II comorbidity is surprising, given the fact that BP-II seems to be the most common bipolar phenotype in patients treated for major depression and/or PD [18,31].

Several studies [32,33] have suggested that BP and PD share common familial and genetic factors. In a follow-up study, MacKinnon *et al. *[32] reported an unusually high prevalence of PD in 57 families of probands with BP. A linkage to markers on the long arm of chromosome 18 was observed only in families of BP probands with comorbid PD. In a recent study [33] on first-degree relatives (n = 966) of probands with bipolar I disorder (n = 192) and schizoaffective disorder, bipolar type (n = 11), more than 90% of subjects with PD also had an affective disorder, and PD was present in 17% of the relatives with recurrent affective disorder compared with 3% of the relatives without recurrent major affective disorder. These findings are consistent with the hypothesis that familial BP increases the risk for PD and this latter may be an index of genetic heterogeneity in BP.

The association between PD and BP also raises important questions from clinical and therapeutic points of view. Most information in the literature is on patients with BP-I and comorbid PD: they have more frequent mixed symptomatology and suicidality, early onset panic attacks, and significant increases in drug abuse and in physical morbidity [1,17,20,34]. Moreover, history of panic attacks in BP proved to be a significant correlate of non-remission; type I BP patients with comorbid PD often require a greater number of medications, either sequentially or in combination, in order to achieve remission [17]. Less information is available on patients with PD and comorbid BP-II; open reports indicated that they are more difficult to treat than patients without such comorbidity [23,25,31].

In order to explore the clinical and therapeutic implications of comorbid BP-II, we analyzed data concerning 326 consecutive patients who sought medical help for PD and who were treated according to routine clinical practice and followed-up for a 3-year period.

## Materials and methods

### Study sample

The sample comprised 326 consecutive outpatients with PD-agoraphobia evaluated and treated at the Psychiatric Institute of the University of Pisa, Italy, from 1991 to 1995, and followed for a period of 3 years. The major aim of the study was to describe the course and the evolution PD-agoraphobia in a setting of routine clinical practice. The study constitutes an offshoot from the Pisa-Memphis (now San Diego) collaboration on the phenomenology and outcome of affective and related disorders.

Inclusion criteria for the present protocol were (1) a principal diagnosis of PD with or without agoraphobia according to Diagnostic and Statistical Manual of Mental Disorders 3rd edn, revised (DSM-III-R) criteria, (2) absence of severe physical and laboratory abnormalities and (3) absence of current psychotic disorders (last 6 months). Previous psychotic features during depressive episodes or depressive mixed states did not represent exclusion criteria. All patients gave informed consent for their participation in the study.

All patients were evaluated by the senior psychiatrists on the project (GP and CT) to ensure that admission criteria were met. Presence of a current or past history of mood or other anxiety disorders (lifetime comorbidity) was not considered as exclusion criteria to ensure the selection of patients that covered the full range of the clinical universe applying for treatment.

The mean age of our sample at the time of admission into the study was 34.5 (SD = 11.5, range = 18–73) and 222 (68.1%) were female. Mean age at onset was 29.3 (SD = 10.7, range = 6–64) for PD and 30.1 (SD = 9.2, range = 14–58) for agoraphobia. Agoraphobia was present in 92.6% (n = 302) of the sample.

### Methods

An intensive face-to-face interview that consisted of structured and semi-structured components was used to collect data. The interview lasted approximately 1 h at baseline, and 0.5 h for subsequent visits. The interviews were performed by senior residents with extensive clinical experience in the diagnosis and treatment of anxiety disorders. Each interviewer underwent a training program in the use of the interview instruments that included direct observation of experienced interviewer, and inter-rater trials. The interviewers were not involved in treatment decisions, which were entrusted to an independent clinician.

At baseline patients were evaluated by means of the Structured Clinical Interview for Diagnosis (SCID) [35], the Panic Disorder/Agoraphobia Interview [36,37] and the Longitudinal Interview Follow-up Examination (Life-Up) [38]. Life-Up has been designed to be administered every 6 months; however, as accuracy is duly enhanced by shorter intervals, it can be administered more frequently according to the specific design of a given study. In our case, it was administered during the periodic visits at every 2 months or at shorter intervals, as dictated by clinical necessity. Patients who interrupted follow-up assessment were contacted at the end of the 3 years of follow-up and evaluated by means of a semi-structured interview, widely utilized in the World-Wide Upjohn Follow-up Study [39]. This particular interview lasted 30–40 min and was carried out face to face or by phone.

High reliability for both principal (k = 0.96) and comorbid (kappa from 0.80 to 0.93) diagnoses have been documented in a subsample (n = 15).

### Panic Disorder/Agoraphobia Interview

This instrument is subdivided into different sections exploring: (1) demographic characteristics, based on the Adult Demographic and Personal Inventory [40]; (2) family history of anxiety, mood and other disorders in first degree relatives, based on Winokur's approach as incorporated into the Family History version of the Research Diagnostic Criteria [41]; (3) personal history of the patient using the first panic attack as the primary anchoring point. Once the period of the first panic attack was described, the number of years preceding and following this event was reviewed with particular focus on the symptomatological characteristics of PD, the course of the illness and comorbidity with other mental disorders; and (4) affective temperaments according to Akiskal and Mallya [42] criteria and avoidant and dependent personality disorders according to DSM-III-R criteria, the former now shown to have good reliability and internal consistency in the Temperament Evaluation of Memphis, Pisa, Paris and San Diego, Interview version (TEMPS-I) [43], and the latter based on the corresponding sections of the SCID [44].

### Life-Up

This is a semi-structured interview and rating system for assessing the longitudinal course of psychiatric disorders in sufficient detail to enable researchers to date individual episodes of any psychiatric disorder and thus to provide the basis for precise calculation of time to recovery, length of ensuing wellness intervals, and time to subsequent relapse or recurrence. The instrument [38] consists of different sections geared to assess psychopathological features, obtain history for psychiatric treatment and psychosocial functioning, as well as that for non-psychiatric medical illness. Finally, the Life-Up provides a global assessment scale according to which the overall health and functioning of the patients are indicated. PD and agoraphobia severity is recorded week by week on a score scale ranging from 1 to 6.

According to this instrument, 'remission' is defined as a period of 8 weeks during which patients with PD have no attacks, though sometimes they may feel on the verge of an acute attack. 'Recurrence' is defined as a period of at least 4 weeks during which patients have one or more panic attacks per day, or a persistent fear of them. For agoraphobia, 'remission' is defined as the absence of avoidance for a period of 8 weeks, while 'recurrence' defines a period of at least 4 weeks, during which avoidance is present.

### Drop-out Interview

This instrument is specifically designed to explore symptoms, disability, help seeking and medications after the end of the study and in the last year [39]. After the registration of the reasons of interruption, such as remission, inefficacy of the treatment, side effects, distance to the clinic, onset of other medical diseases, etc., the course of the illness was investigated. In this way, remissions and recurrences throughout the period from the interruption of the study to the moment of the interview were recorded. Moreover, drugs that had been taken during that period were registered.

### Treatment management

Clinicians involved in the treatment decisions and management were independent from the raters. Patients were treated according to routine clinical practice with antidepressants. Most of them received imipramine (n = 127, 39%), clomipramine (n = 93, 28.5%) or paroxetine (n = 76, 23.3%). Only a few patients (n = 30, 9.2%) were assigned to other medications, such as fluvoxamine, fluoxetine, citalopram or trimipramine, when, according to the individual physician's judgment, it was not possible to treat them with the former drugs, either because of hypersensitivity or previous negative experiences.

In the first years of the study, tricyclic antidepressants (TCAs) represented the best known antipanic agents and for this reason they have been widely employed in our patients. Subsequently, the acquisition of the antipanic properties of paroxetine [45] has prompted the use of these new compounds in the outpatients enrolled in the follow-up study. Only since 1995 has paroxetine emerged in our experience as a viable first line intervention drug for PD-agoraphobia. Initially, paroxetine was prescribed to patients judged less severe. Subsequently, paroxetine was also prescribed to those subjects for whom TCAs were contraindicated, as well as in those who had shown a jitteriness syndrome or supersensitivity to anticholinergic side effects. The initial dose was for 10 mg of imipramine or clomipramine and 10 mg of paroxetine. Dosage was gradually increased (10 mg every 2 days for TCAs and 5 mg every 4 days for paroxetine) to a maximum of 300 mg and 40–50 mg respectively. The treating clinician could raise or lower the dose, depending upon the individual patient's clinical state or side effects. After the first 4 weeks of treatment and during each subsequent interval assessed, most patients remained on nearly the same dose of antidepressant. At the fourth week, the daily mean dosages of imipramine, clomipramine and paroxetine were, respectively, 132.6 ± 74.3 mg/day (range 30–250), 109.3 ± 84.6 mg/day (range 40–300), and 28.7 ± 14.6 mg/day (range 10–60). Superposable values were observed at the beginning of the first remission (imipramine, 116 ± 79.8 mg/day, range 30–300; clomipramine, 126.2 ± 73.8 mg/day, range 25–250; paroxetine, 30.6 ± 11.2 mg/day, range 10–50) and at the last evaluation (imipramine, 121.8 ± 81.2 mg/day, range 30–300; clomipramine, 115.9 ± 74.4 mg/day, range 25–250; paroxetine, 28.6 ± 10.2 mg/day, range 10–50). No statistically significant differences in daily mean dose of antidepressants were observed between patients with or without bipolar II comorbidity.

At baseline, for those patients who were taking benzodiazepines (BDZ) a gradual tapering was scheduled. Subsequently, the administration of BDZ was allowed only occasionally when absolutely unavoidable.

When comorbidity with BP-II was present, mood stabilizers such as valproate or lithium were prescribed. The latter was based on clinicians' judgment, according to routine clinical practice. All bipolar patients were deemed by the investigators to have derived maximum benefit from the treatment with mood stabilizers prior to receiving antidepressants. At the time the latter were started, PD/agoraphobia was present at a clinical level in all subjects.

### Statistics

Statistical comparisons between patients with PD (n = 274) and PD+BP-II (n = 52) were conducted by means of analysis of variance (ANOVA) and chi-square analysis for continuous and categorical variables, respectively. Kaplan-Maier survival analysis was utilized to define the relationship between PD course (length of periods free from the illness and number of recurrences) and the presence of comorbid BP-II.

## Results

Of the 326 PD patients of our sample, 52 (16%) had comorbid BP-II. At the time that treatment with antidepressants was started, none of them were suffering from hypomanic episode, but a major depressive episode presented in 21 (40.4%) vs 91 (33.2%) subjects, respectively in the group with and without bipolar II comorbidity (chi square = 1.0; p = not significant (NS)).

Comparisons between bipolar patients and those without BP-II showed similar results with respect to demographic and clinical features, such as age, sex distribution, age at onset, length of illness, presence of agoraphobia, personality and temperamental features, as well as the severity scores of PD and agoraphobia and Global Adjustment Score (GAS) at baseline evaluation (Table 1).

**Table 1:** Clinical characteristics of panic disorder patients with and without comorbid bipolar disorder

	**PD (n = 274)**	**PD+BD (n = 52)**	**F/chi-square**	**p Value**
Age, mean (SD)	36.7 (11.5)	35.1 (11.6)	-0.9	NS

Sex, female, n (%)	191 (69.7)	31 (59.6)	2.0	NS

Agoraphobia, n (%)	219 (79.9)	44 (84.6)	0.06	NS

First degree family history, n (%):				

Mood disorders	83 (30.3)	23 (44.2)	3.9	0.05

Substance abuse	10 (3.7)	3 (5.8)	0.5	NS

Panic-agoraphobic disorder	110 (40.1)	33 (63.5)	9.6	0.002

Age at onset, mean (SD)	29.9 (10.5)	26.0 (11.0)	-2.5	NS

Length of illness in months, mean (SD)	87.2 (128.4)	117.4 (159.4)	1.5	NS

Life-up scores at baseline, mean (SD):				

Panic disorder	3.4 (1.0)	3.2 (0.8)	-0.9	NS

Agoraphobia	3.0 (1.2)	3.0 (1.0)	0.2	NS

General adjustment (GAS)	69.8 (11.1)	68.3 (9.8)	-0.9	NS

Lifetime comorbidity, n (%):				

Major depression	91 (33.2)	-		

Generalized anxiety	46 (16.8)	12 (23.1)	1.2	NS

Social phobia	21 (7.7)	9 (17.3)	4.9	0.03

Obsessive-compulsive	20 (7.3)	11 (21.1)	9.7	0.002.

Alcohol-related disorders	16 (5.8)	9 (17.3)	8.1	0.004.

Separation anxiety	85 (31.0)	24 (46.1)	4.5	0.03

Personality disorders, n (%):				

Avoidant	64 (23.4)	16 (30.8)	1.3	NS

Dependent	37 (13.5)	7 (13.5)	0.000	NS

BP, bipolar disorder; GAS, Global Adjustment Score; NS, not significant; PD, panic disorder.

Some statistically significant differences were observed as regards the lifetime presence of other comorbid mental disorders: patients with BP-II showed a significantly greater frequency of social phobia and obsessive-compulsive disorder, as well as alcohol-related disorders, in comparison with patients without bipolar comorbidity. Bipolar patients were also significantly more likely to report a positive history of separation anxiety during childhood and adolescence. The presence of comorbid generalized anxiety disorder was not significantly different between the two groups (Table 1).

As regards family history, a significantly greater frequency of PD-agoraphobia was present in the group with BP, as well as a greater familial load for mood disorders. Family history of substance-related disorders (including alcohol) was similar in the two groups (Table 1).

No significant differences were observed in the efficacy of the various drug treatments (Table 2). Likewise, the comparison between bipolar and non-bipolar PD patients showed that the presence of BP-II did not influence the likelihood of staying in the follow-up or interrupting the pharmacological treatment for various reasons, such as remission or ineffectiveness (Table 3). Moreover, the presence of comorbidity with BP-II did not appear to have significant effects on the long-term course of either PD or agoraphobia. Kaplan-Maier survival analysis showed that time of remission both for PD (Mantel-Cox chi-square = 0.018, df = 1, p = NS) and agoraphobia (Mantel-Cox chi-square = 0.002, df = 1, p = NS) was similar in the two groups. Also similar was the time of relapse for PD (Mantel-Cox chi-square = 0.963, df = 1, p = NS) and for agoraphobia (Mantel-Cox chi-square = 0.969, df = 1, p = NS). Additionally, no difference between patients with and without comorbid BP-II was observed in the mean duration of total remission and relapse periods (Table 4).

**Table 2:** Drug treatments of panic disorder patients with and without comorbid bipolar disorder

	**PD n (%)**	**PD+BD n (%)**	**Chi-square**	**p Value**
Imipramine	105 (38.3)	22 (42.3)		
Clomipramine	81 (29.6)	12 (23.1)		
Paroxetine	60 (21.9)	16 (30.8)		
Other	28 (10.2)	2 (3.8)	4.2	NS

BP, bipolar disorder; NS, not significant; PD, panic disorder.

**Table 3:** Different reasons of treatment interruption in PD patients with and without comorbid bipolar disorder

	**PD n (%)**	**PD+BD n (%)**	**Chi-square**	**p Value**
In treatment	122 (44.5)	25 (48.1)		
Remission	56 (20.4)	10 (19.2)		
Ineffectiveness	45 (16.4)	7 (13.5)		
Lost to follow-up	41 (15.0)	7 (13.5)		
Other reasons	10 (3.6)	3 (5.8)	0.9	NS

BP, bipolar disorder; NS, not significant; PD, panic disorder.

**Table 4:** Long term course in PD patients with and without comorbid bipolar disorder

	**PD, mean (SD)**	**PD+BD, mean (SD)**	**F/chi-square**	**p Value**
PD:				
Remission length, months	72.2 (40.9)	73.4 (39.6)	0.2	NS
Relapse length, months	26.0 (22.2)	28.4 (25.6)	0.5	NS
Agoraphobia:				
Remission length, months	72.5 (42.6)	78.1 (40.6)	0.8	NS
Relapse length, months	29.3 (23.8)	32.0 (25.9)	0.4	NS

BP, bipolar disorder; NS, not significant; PD, panic disorder.

## Discussion

This study evaluates the prevalence of BP-II lifetime comorbidity and its consequences on clinical features and treatment response in a large group of PD outpatients followed in a naturalistic setting of routine pharmacological treatment. To our knowledge, this is the first study that has dealt with the impact of BP-II on the course of PD in patients selected on the basis of PD-agoraphobia as a principal diagnosis. The 16% prevalence rate of comorbid BP-II in our patients is consistent with those observed in other clinical samples selected with similar criteria [19,23,25]. The findings from different psychiatric centers in both Europe and the US go against a common perception that the relationship between anxiety and mood disorders is largely limited to unipolar depression and dysthymia. The importance of screening all PD-agoraphobic patients for past hypomania should be emphasized.

We did not find any significant effect of BP-II comorbidity on the clinical features of PD, including those related to the severity of the disease such as age at onset of panic attacks, baseline severity of PD-agoraphobia, general adjustment scores, and length of illness. Moreover, such comorbidity does not appear to have significant effects on the long-term outcome of the PD-agoraphobia, as indicated by the mean duration of the remission or the relapse periods. Even the responses to pharmacological therapy and the patients' adherence to long-term drug treatment do not appear to be significantly influenced by the presence of BP-II.

Previous clinical studies on BP-I patients with comorbid PD showed more severe symptomatology with early onset of panic attacks. Moreover, they were more difficult to treat than BP subjects without comorbid PD [8,17,23,34,46]. This discrepancy might be accounted for by the different criteria used for the selection of the sample. In fact, whereas other clinical studies typically described inpatients with mania and/or mixed mania with concomitant PD, the present study focused on PD outpatients with comorbid BP-II. This choice was justified by growing evidence that limiting the diagnosis of the bipolar spectrum disorders to the classical type I form is too restrictive and severely underestimates the presence and impact of the bipolar disease [2,47,48].

Moreover, the present study aimed to investigate the presence and the implications of bipolar comorbidity on PD-agoraphobia in a naturalistic setting of routine clinical practice, and BP-II is the most common form of bipolarity in such a setting [49]. Broadening the inclusion of bipolarity to less severe, softer and more prevalent forms might account for the milder effects of bipolar comorbidity on the severity and course of PD-agoraphobia that were found in our sample.

BP-II comorbidity, nonetheless, appears to influence the pattern of lifetime clinical features in PD patients, especially with regard to the presence of alcohol and substance use and other concomitant anxiety disorders, such as social phobia and obsessive-compulsive disorders. The coexistence of multiple anxiety and substance-related disorders with bipolarity has been reported in previous clinical studies [48,50,51].

Although the severity and the outcome of panic-agoraphobic symptomatology seem to be similar in patients with and without BP-II comorbidity, the higher number of concomitant disorders in our PD patients with BP-II indicated a greater complexity of the overall clinical picture. The latter might explain the treatment difficulties reported in open clinical observations [23,25,31]. The fact that our center specializes in BP-II may have led to optimal or reasonable stabilization of the bipolar component of the comorbidity in the present sample.

Another interesting finding is the presence of higher rates of childhood separation anxiety among subjects with comorbid BP-II in comparison with other PD patients. Anxious bipolarity thus appears to be related to a more intense vulnerability for early onset phobic-anxious manifestations, indicating that clinical complexity in these patients extends to childhood precursors of PD-agoraphobia [52].

The pattern of complex relationships among PD and mood disorders would require better-designed prospective observations. Nonetheless, the validity of the phenomenon of anxious-bipolar comorbidity should no longer be in doubt. This relationship is further suggested by our finding of a stronger load of PD-agoraphobia among the first-degree relatives of PD subjects with BP-II. Previous reports from family studies have also reported high prevalence rates of PD in families with a high load of bipolar disorder [32,33].

Our findings are consistent with the hypothesis that, at least in a significant minority of cases, PD and BP-II may share common familial and genetic factors, and these factors may influence the earliest manifestations of the panic-agoraphobic syndrome and the complexity of the longitudinal clinical picture. By contrast, bipolar comorbidity does not seem to have significant effects on the severity of the core symptomatological features, as well as the clinical course or response to treatments of the full-blown panic-agoraphobic syndrome that, hypothetically, appears to be shaped by other factors, possibly distinct and unrelated to BP comorbidity.

Other attractive theoretical possibilities must be considered too. Indeed, the recognition of bipolar comorbidity in PD patients has relevant theoretical and practical implications. In hypothesizing a putative common substrate, the fact that not only depression but also (hypo)mania and mixed states frequently coexist with anxious-phobic symptomatology should be taken into account. Hypomanic switches on antidepressants or alcohol represent frequent coexisting mood states in the longitudinal history of many PD patients [1,4,18,53]. In a more theoretical vein, we submit that the foregoing considerations challenge the view that anxiety and bipolar spectrum disorders are completely independent syndromes, and they stress the advantage of diagnosing such comorbidity from the psychopathological, clinical, and public health perspectives. To recapitulate what we have advanced in the present work, the co-occurrence of bipolar and panic-agoraphobic spectra suggests that a distinct anxious-bipolar diathesis does exist. Most panic-agoraphobic disorders are probably unrelated to such an entity. By contrast, a substantial proportion of BP-II might belong to the joint anxious-bipolar diathesis, possibly part of an even broader spectrum of psychopathology [48].

One important caveat of this study is that we focused herein on the pharmacologic treatment of the PD-agoraphobia in otherwise mood stabilized BP-II. The optimum treatment of BP-II in the presence of panic-agoraphobic disorders, often also comorbid with social phobia, represents a therapeutic challenge to clinicians. Such panic-agoraphobia comorbidity has been described with BP-I, with delayed and poorer naturalization outcome for the bipolar disorder [54]. In our clinical experience, the same is equally true for BP-II, unless it is stabilized before instituting pharmacologic treatments for the anxious comorbidity. This topic will be the subject of a subsequent manuscript by our group.

## Competing interests

The authors declare that they have no competing interests.

## Authors' contributions

CT, GP, KNF, KA and HSA conceived of the study, and participated in its design. CT and GP coordinated the study. FF and GT evaluated all the patients under the supervision of CT and GP. GP performed the statistical analysis. All authors read and approved the final manuscript.
